# Dynamic alterations in the paternal epigenetic landscape following fertilization

**DOI:** 10.3389/fgene.2012.00143

**Published:** 2012-07-31

**Authors:** Timothy G. Jenkins, Douglas T. Carrell

**Affiliations:** ^1^Andrology and IVF Laboratories, Department of Surgery, University of Utah School of Medicine,Salt Lake City, UT, USA; ^2^Department of Obstetrics and Gynecology, University of Utah School of Medicine,Salt Lake City, UT, USA; ^3^Department of Physiology, University of Utah School of Medicine,Salt Lake City, UT, USA

**Keywords:** fertilization, epigenetics, chromatin, embryogenesis, DNA methylation

## Abstract

Embryonic development is a complex and dynamic process with frequent changes in gene expression, ultimately leading to cellular differentiation and commitment of various cell lines. These changes are likely preceded by changes to signaling cascades and/or alterations to the epigenetic program in specific cells. The process of epigenetic remodeling begins early in development. In fact, soon after the union of sperm and egg massive epigenetic changes occur across the paternal and maternal epigenetic landscape. The epigenome of these cells includes modifications to the DNA itself, in the form of DNA methylation, as well as nuclear protein content and modification, such as modifications to histones. Sperm chromatin is predominantly packaged by protamines, but following fertilization the sperm pronucleus undergoes remodeling in which maternally derived histones replace protamines, resulting in the relaxation of chromatin and ultimately decondensation of the paternal pronucleus. In addition, active DNA demethylation occurs across the paternal genome prior to the first cell division, effectively erasing many spermatogenesis derived methylation marks. This complex interplay begins the dynamic process by which two haploid cells unite to form a diploid organism. The biology of these events is central to the understanding of sexual reproduction, yet our knowledge regarding the mechanisms involved is extremely limited. This review will explore what is known regarding the post-fertilization epigenetic alterations of the paternal chromatin and the implications suggested by the available literature.

## INTRODUCTION

Embryogenesis is a dynamic and complex process that begins with the union of oocyte and sperm, where each gamete contributes their haploid genome and cellular content to the developing zygote. To successfully navigate embryonic development specific epigenetic cues must exist in both the paternal and maternal chromatin to drive activation or silencing of various genes or gene families, ultimately resulting in cellular differentiation. These epigenetic marks can be found on the DNA, as with 5-methylcytosine (5-mC) and 5-hydroxymethylcytosine (5-hmC), or in the form of various modifications on histone tails, and are sufficient to regulate gene activation both independently or in concert with each other.

In many somatic cell types, the effects of epigenetic marks on the function of cell activities and pathologies is relatively well characterized. Similarly, much is known regarding the importance of the epigenome in sperm (Biermann and Steger, 2007; Hammoud et al., 2009; Jenkins and Carrell, 2010; Navarro-Costa et al., 2010; Puri et al., 2010). However, the complex interplay and rearrangements of various epigenetic marks during embryonic development are not well defined, mainly due to the difficulty in studying early embryos and the dynamic nature of the epigenome immediately following fertilization. Embryonic development is extraordinarily dynamic and results in the generation of multiple cell types by varying gene activation or silencing, which is likely preceded, at least in part, by epigenetic modifications.

Knowledge of the epigenetic changes that occur in both the paternal and maternal chromatin post-fertilization and the roles of these modifications in development is essential to understanding the process of embryogenesis. A more complete understanding of the epigenetic modifications that drive embryonic development will provide important insight into many fields of research and will aid in the development of important clinical applications, including stem cell biology, reproductive medicine, and likely, pediatric and adult health. This review will focus specifically on the paternal epigenetic landscape in the mature sperm and the modifications that occur post-fertilization. Additionally, the importance of paternal epigenetic marks in the developing embryo will be discussed.

## THE EPIGENETIC LANDSCAPE IN MATURE SPERMATOZOA

The epigenome of the mature sperm is specialized to facilitate its unique role. Nuclear proteins found in the sperm form a distinct chromatin structure that is unlike any other cell type and is perfectly suited to support the male gamete. The utility of this highly specialized epigenome is to facilitate the safe delivery of competent paternal DNA required to, in concert with the maternal genome, generate a viable offspring. Protamine proteins are the most abundant nuclear proteins found in sperm and are unique to sperm cells. These proteins have a strong positive charge due to their high arginine content, which helps facilitate their function (Oliva and Dixon, 1990; Dadoune, 1995). During the process of spermatogenesis protamines replace 85–95% of histones in the sperm, including both canonical histones and testicular histone variants, via a stepwise process. First, transition proteins, comprising both transition proteins 1 and 2 (T1 and T2), replace histone proteins that are DNA bound. Second, T1 and T2 are replaced by protamine proteins, protamine 1 and protamine 2 (P1 and P2). The ratio of P1:P2 is approximately 1:1 in most fertile humans (Balhorn et al., 1988; Aoki et al., 2006b; Nanassy et al., 2011).

Once protamines are incorporated into the paternal chromatin, cysteine residues between protamine molecules form intermolecular disulfide bridges as the cell matures. The strong positive charge of P1 and P2 as well as the formation of disulfide bridges produces a tightly condensed chromatin structure. In fact, the protamine bound DNA in sperm is approximately 6–20 times more dense than a nucleosome bound chromatin structure (Ward and Coffey, 1991; Balhorn, 2007). The increased density of the mature sperm is believed to play two main roles. First, the motility of sperm is thought to rely on a condensed nuclear structure, as a decondensed sperm head may mechanically inhibit or perturb the cell’s potential for motility (Carrell and Hammoud, 2010). Second, mature sperm lack robust DNA repair mechanisms, and thus DNA damage must be prevented. A tightly condensed chromatin structure provides significant protection from the DNA damage that could arise in the male and female reproductive tract prior to fertilization (Aoki et al., 2006a).

Although this unique nuclear structure provides what is requisite for appropriate sperm function in the mature gamete, it also creates a quiescent chromatin largely void of valuable epigenetic regulatory marks in the form of histone tail modifications. These modifications typically occur at lysine or serine residues on the histone tail and include methylation, acetylation, ubiquitination, and phosphorylation and are known to exert potent epigenetic regulation in various cell types (Lachner and Jenuwein, 2002; Suganuma and Workman, 2008). However, in the mature sperm the protamination process replaces the majority of histone proteins with protamines to aid in chromatin compaction (Ward and Coffey, 1991). In the process, regulatory marks, in the form of histone tail modifications, are removed to achieve the more pressing need of DNA protection and sperm motility.

Until recently, the quiescent, largely protamine bound nature of sperm chromatin lead researchers to subscribe to the theory that the role of the sperm epigenome in embryogenesis was limited at best. However, it has been demonstrated that the replacement of histones in the developing sperm is an incomplete process, leaving approximately 5–15% of the genome bound by nucleosomes (Tanphaichitr et al., 1978; Wykes and Krawetz, 2003). In fertile donors this nucleosome retention was determined to be programmatic in nature and followed a pattern in which histones were retained at loci important in embryonic development (Arpanahi et al., 2009; Hammoud et al., 2009). These data established a possible role for paternal epigenetic marks not only in the developing and mature sperm, but also in early embryogenesis.

## EVIDENCE SUGGESTING A ROLE FOR THE PATERNAL EPIGENOME IN EMBRYOGENESIS

Many types of epigenetic marks are important in the regulation of gene expression and thus cell function. Included among these various epigenetic marks in sperm are histone tail modifications, programmatic histone retention (following the process of protamination), DNA methylation, and the formation of DNA demethylation intermediates. Perturbations in these epigenetic marks have been associated with poor spermatogenesis and thus decreased fertility, poor fertilization ability, embryo quality, and even pregnancy outcome (Doerksen and Trasler, 1996; Kelly et al., 2003; Lee et al., 2005; Aoki et al., 2006b; Oakes et al., 2007; Glaser et al., 2009). Additionally, gynogenetic mammalian embryos (as well as androgenetic) generated via pronuclear transplant are unable to complete embryogenesis and thus cannot generate viable offspring (McGrath and Solter, 1984; Surani et al., 1984). Taken together these data demonstrate that the paternal epigenome is required not only for spermatogenesis and mature sperm function, but for embryonic development as well.

Many mammalian studies suggest an important role of the paternal epigenome in early embryonic development. Various knock out or knock down mouse models for DNA methyltransferase (DNMT) proteins have demonstrated their essential role in sperm DNA methylation and overall male fertility (Kaneda et al., 2004; Kato et al., 2007; La Salle et al., 2007) but few have addressed the pregnancy outcomes from mating these animals. Other murine studies have utilized pharmaceutical approaches to alter methylation patterns in the male germ cell and have observed altered pregnancy outcomes. Seven-week-old male mice treated with 5-aza-2′-deoxycytidine, a potent *de novo* DNA methylation inhibitor, display significant global hypomethylation in sperm. When these animals were mated with virgin 8-week-old females they displayed significantly decreased pregnancy rates and increased pre-implantation loss (Kelly et al., 2003). Similar results were found in rat studies where males were treated with 5-azacytidine. Embryos sired by male rats treated with 5-azacytidine displayed decreased embryo quality and increased pre-implantation loss (Doerksen and Trasler, 1996). Although measures were taken to ensure that the effects on pregnancy outcome were due to DNA methylation alterations (by utilizing control treatments from the azacytidine family members that do not affect DNA methylation) it should be noted that some of these effects may be a result of the cytotoxic affects of the pharmaceutical agents and not completely isolated to DNA methylation aberrations. Recent data from human subjects undergoing *in vitro* fertilization (IVF) likewise suggests that altered DNA methylation can impact embryo development. In a recent study 63 individuals provided sperm samples that were analyzed for global DNA methylation status. It was found that global sperm DNA hypomethylation is associated with poor pregnancy outcomes (Benchaib et al., 2005). Taken together these data show the importance of sperm DNA methylation in embryonic development.

Recent data also support a role for paternal epigenetics in embryogenesis. Hammoud et al. (2011) found that patients with unexplained poor embryo quality did not have the typical distribution of retained histones that is seen in fertile men. In fact, the retention appeared to be random and not programmatic. Additionally a marked decrease in the enrichment of modified histones, H3 lysine 4 methylation (H3K4me) and H3 lysine 27 methylation (H3K27me), at important developmental loci was observed. It has also been suggested that poor embryo quality, which is often seen in mouse round spermatid injection is at least in part due to the immature epigenetic landscape in these cells (Kishigami et al., 2006). Aberrant DNA methylation status in the paternal nucleus and an inability to drive proper DNA methylation reprogramming in the embryo were commonly noted in cases of round spermatid injection (Kishigami et al., 2006). The body of evidence suggests that while the oocyte and its machinery are essential to the process of chromatin remodeling, there is epigenetic regulation that is facilitated by the sperm and/or the paternal chromatin. Although much work is still needed to fully elucidate the role of the paternal epigenome in this process some important stages of post-fertilization chromatin remodeling have been described and provide background for future research in the field.

## PROTAMINE TO HISTONE TRANSITION POST-FERTILIZATION

Following fertilization, the highly compacted sperm chromatin must be reorganized from its highly compacted and transcriptionally quiescent state to an inducible state to facilitate the needs of the zygote and early embryo. In addition to the resumption of the oocyte’s cell cycle, the remodeling of the paternal chromatin is one of the key events that occurs post-fertilization (McLay and Clarke, 2003). Although it is known to be a key step in the activation of the paternal pronucleus, the timing and regulation of protamine removal and replacement by maternally derived histones is poorly understood. This event is difficult to study in all mammals, and in particular humans, due to ethical and technical restrictions.

**FIGURE 1:**
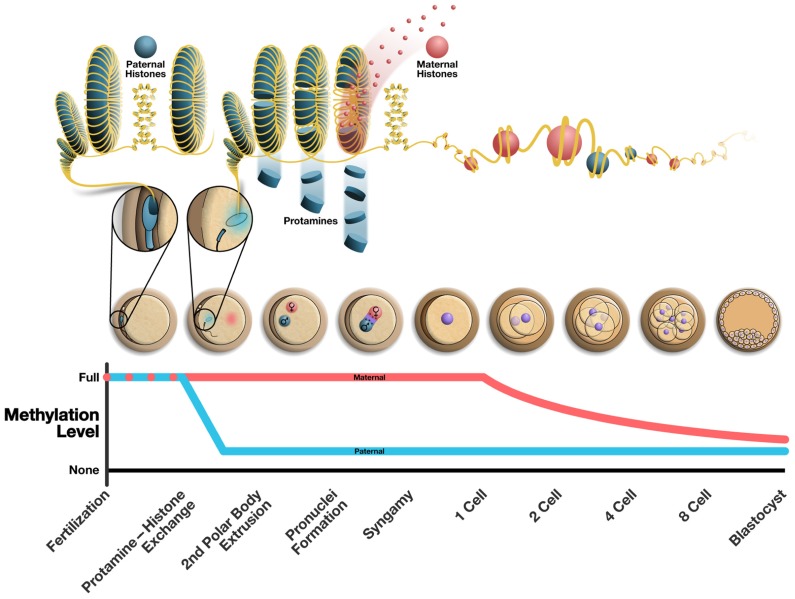
**Alterations to the epigenome post-fertilization.** The top panel illustrates the chromatin structure of the mature sperm immediately following fertilization (highly protaminated with some retension of paternally derived histones). From the following cell is seen the protamine to histone transition where maternally derived histones replace protamines resulting in the decondensation of the sperm head. The middle panel illustrates the various stages of early embryonic development. The bottom panel shows the methylation changes that occur over time in the maternal and paternal pronucleus, where the paternal pronucleus undergoes active demethylation and the maternal DNA is demethylated passively in a replication dependent manner. The approximate chronology of major events in the early embryo is outlined along the bottom of the figure and correlates to the illustrations of embryos above.

As a result of the difficulties in studying epigenetic remodeling of the paternal chromatin early in embryogenesis most data regarding protamine replacement are derived from various mammalian model studies or from human sperm with heterologous intracytoplasmic sperm injection (ICSI). Because of the high degree of compaction, remodeling the mature sperm chromatin post-fertilization is essential to generating a transcriptionally competent DNA that can contribute to embryonic development. This process must occur prior to DNA replication and mitosis (Nonchev and Tsanev, 1990). Following fertilization sperm DNA is decondensed and expands to approximately three times the size of the mature sperm nucleus, resulting in the formation of the paternal pronucleus (**Figure 1**). These events are thought to coincide with the complete removal of protamine from the paternal chromatin (Wright and Longo, 1988; Adenot et al., 1991; Perreault, 1992; McLay and Clarke, 2003; Jones et al., 2011). The timing of protamine removal, histone incorporation, chromatin decondensation, and the generation of the paternal pronucleus has been investigated, but results remain controversial. In porcine studies of IVF it was determined that the protamine to histone transition occurred between 2 and 4 h post-fertilization. The majority of protamines (approximately 80%) were removed within 3 h, at which point histone association with DNA begins and is completed by approximately 4 h after fertilization (Shimada et al., 2000; Nakazawa et al., 2002). It was also noted that both protamine removal and histone incorporation occurred prior to full decondensation and male pronucleus formation in the pig.

In mouse studies the reported timing of protamine removal varies from nearly immediately after oocyte penetration to as late as 8 h post-fertilization (Rodman et al., 1981; Nonchev and Tsanev, 1990). In a recent study on human sperm following heterologous ICSI with hamster ova, the protamine removal was completed within 1 h of ICSI with significant zygote to zygote variability (Jones et al., 2011). The timing of protamine removal post-fertilization remains controversial and poorly characterized, but it is clear that this process must be completed prior to paternal DNA replication and transcription and as such must occur soon after fertilization.

The oocyte plays an important role in the removal of protamine proteins from the paternal chromatin. Based on bovine and hamster studies, protamine removal is believed to rely on reduction of disulfide bonds between proteins via the activity of maternally derived glutathione (Perreault et al., 1988; Sutovsky and Schatten, 1997). The role of this important antioxidant in breaking disulfide bonds and facilitating the decondensation of paternal chromatin is further supported by the observation of increasing levels of glutathione as mammalian eggs mature, with coinciding increases in chromatin relaxation (Perreault et al., 1988). Interestingly, a common hypothesis of chromatin compaction in the maturing spermatid requires a unique sperm nuclear form of glutathione peroxidase to facilitate disulfide bridge formation (Pfeifer et al., 2001; Bertelsmann et al., 2007). It appears that glutathione and glutathione peroxidase may be key to both compaction of the nucleus and relaxation post-fertilization. Although concrete mechanisms for the replacement of protamines with histones post-fertilization have not been characterized it is clear that the decondensation of sperm chromatin is essential. This process requires further study to determine the role of the paternal chromatin in the early embryo and the regulation that is accomplished by the oocyte. An improved understanding of these mechanisms result in improved diagnosis and treatment of patients undergoing assisted reproductive therapies such as IVF.

### DYNAMICS OF POST-FERTILIZATION DNA METHYLATION

The epigenomic landscapes of maternal and paternal gametes are distinct from one another and from somatic cells. For the union of gametes to result in the generation of viable offspring, fertilization must induce the resumption of the cell cycle in the oocyte, generate a diploid cell line through syngamy, and re-establish an epigenetic state appropriate for embryonic development. This requires massive reprogramming of many epigenetic marks in both the paternal and maternal pronuclei soon after fertilization, followed by further reprogramming to direct embryonic development (Reik et al., 2001; Li, 2002). Among the many epigenetic changes occurring soon after fertilization, one of the most striking is “global” DNA methylation erasure in the paternal pronucleus, which effectively removes most methylation marks across the paternal genome (Abdalla et al., 2009a). However, the term global is often misused in describing this event, as there are distinct regions in the paternal genome that escape demethylation. Specifically, these regions include imprinted clusters and retrotransposons (Abdalla et al., 2009a; Hales et al., 2011). This dramatic demethylation process must take place to remove gamete specific regulatory marks, which were established to facilitate sperm function, and allow the laying down of marks competent to direct embryogenesis (Reik et al., 2001; Meehan, 2003).

The active demethylation of the paternal pronucleus is in stark contrast to the passive, replication dependent reduction in DNA methylation that occurs in the maternal genome over successive cellular divisions (Rougier et al., 1998; Mayer et al., 2000; Young and Beaujean, 2004; **Figure 1**). There is evidence in mammalian cells that suggests a role for both repressive histone modifications unique to the maternal chromatin and/or the localization of PGC7/Stella (a maternally derived factor important in development) in the maternal pronucleus to guard against demethylation, but this requires further investigation (Nakamura et al., 2007; Hajkova, 2010). To date the process of active demethylation in the paternal DNA is poorly understood. Many factors have been targeted as likely candidates essential to this process but concrete mechanisms for the active removal of DNA methylation marks remain elusive. The DNA demethylase activity of multiple candidate proteins, including growth arrest and DNA damage-inducible protein 45 alpha (GADD45α) and methyl-binding domain protein-2 (MBD2), has been established (Bhattacharya et al., 1999; Barreto et al., 2007). However, neither GADD45 nor MBD2 were found to be essential in the active demethylation of the paternal pronucleus.

Interestingly, recent data suggest that our inability to find key enzymes in the process of active demethylation may be due to a more complex process than was previously expected. Recently, Wossidlo et al. (2010) found that the various phases of DNA demethylation are concomitant with DNA strand breaks and thus DNA repair. These data support previous studies that have suggested an important role for various forms of DNA repair in the process of demethylation (Razin et al., 1986; Weiss et al., 1996). Additional studies have suggested the possibility of using DNA repair mechanisms to repair T:G mismatches, by MBD4, resulting from targeted deaminase conversion of 5-mC into thiamine, and ultimately leaving the DNA in a demethylated state (Hendrich et al., 1999; Morgan et al., 2004; Abdalla et al., 2009b). Even the most prominent proteins involved in *de novo* methylation, DNMTs, have been proposed as possible facilitators of demethylation (Metivier et al., 2008). These data suggest that there is an association between DNA repair and DNA demethylation in the paternal pronucleus, and that there is likely no single protein, or family of proteins responsible for this dramatic event. This may be due to a large degree of functional redundancy between the candidate proteins. Ultimately the result in both the paternal and maternal pronuclei is the early erasure of the majority of gamete derived DNA methylation marks, which provides a relatively blank slate for the establishment of tissue specific methylation patterns required for embryonic development.

The timing of paternal demethylation post-fertilization has not been fully elucidated. One reason for this is the high degree of variability between species and between techniques used to generate offspring, in addition to contradictory reports. The mouse model provides a good benchmark for complete demethylation of the paternal pronucleus and is typically completed within 10 h post-copulation (Zaitseva et al., 2007). In rat, the relative demethylation was reported to be either less than that in the mouse (Zaitseva et al., 2007) or fully demethylated as in the mouse and this process was completed by 16 h post-copulation (Dean et al., 2001; Zaitseva et al., 2007). A greater number of reports of demethylation timing and completion are available for IVF embryos. In mouse demethylation is complete within 4 h after fertilization while in rat and bovine methylation erasure is accomplished by 10 h post-IVF (Mayer et al., 2000; Santos et al., 2002; Abdalla et al., 2009a). The time to complete demethylation in bovine was reduced to 6 h when the embryos were generated with the use of ICSI compared to IVF (Abdalla et al., 2009a). It is difficult to discern the timing of the active demethylation of the paternal nucleus in mammalian zygotes in general due to the high interspecies variability. However, it is clear that this event occurs very early in the developmental process and that it is complete by the first cellular division.

### POST-FERTILIZATION TRANSCRIPTION

The initiation of transcription from both the maternal and paternal DNA following fertilization is difficult to detect and study. The oocyte provides large quantities of previously transcribed RNA to the zygote, which are believed to aid in directing embryogenesis. This native, maternally derived, RNA has also been shown to quickly degrade following fertilization, further complicating the study of transcription initiation in the zygote (Aoki et al., 1997; Kageyama et al., 2004). For both of these reasons characterizing the dynamics of transcription in the zygote has remained elusive. Despite this, some events have been well-established following transcription in some models. Many studies have described a state of transcriptional repression between the two- and four-cell stage in the mouse (Majumder et al., 1993; Henery et al., 1995; Davis et al., 1996). This repressive state has been theorized to be important in preventing globally active DNA with uninhibited transcription, and as a result is essential for normal development (Henery et al., 1995; Ma et al., 2001). It is widely held that transcription initiation is “maternally directed,” that is the machinery from the oocyte is primarily responsible for transcription in the zygote and the early embryo (Evsikov and Marin de Evsikova, 2009). However, it is also clear from recent studies that the paternal epigenome is required to induce the repressive state between the two- and four-cell stage (Bui et al., 2011). By utilizing parthenogenic mouse embryos and those created via ICSI with mature sperm and round spermatids, it was discovered that the paternal epigenome is essential to develop viable offspring (Bui et al., 2011). This study demonstrated that the paternal chromatin is required to aid in silencing the zygotic DNA at the two- to four-cell stage. It was also determined that this ability is acquired throughout spermatogenesis, again suggesting this is a function of the paternal epigenome.

Within the first few rounds of cell divisions in the embryo, some components of the epigenome are known. Early in zygotic development, at the early two-cell stage, small amounts of microRNAs (miRNAs) are transcribed, and by the eight-cell stage in mouse, zygotically derived miRNAs begin to appear in larger quantities, while other gamete derived small RNAs (siRNAs and piRNAs) are degraded (Yang et al., 2008; Ohnishi et al., 2010). It is believed that these miRNAs work in concert with the recently established DNA methylation marks and histone modifications to provide the appropriate epigenetic landscape for embryogenesis. The classification of transcriptional activity at varying stages of pre-implantation embryogenesis is needed to increase our understanding of the mechanistic biology behind this process. Additionally, this knowledge may lead to the development of improved embryo selection techniques and thus improved outcomes for those undergoing assisted reproductive therapies.

### THE ROLE OF PATERNALLY DERIVED RNAs

Recently there has been increased interest in the classification and role of RNAs that are accumulated throughout spermatogenesis and exist in an inert state in the mature sperm. Ostermeier et al. (2002) classified a set of spermatozoal RNAs that were found consistently in fertile men. The discovery of these full length, in tact RNAs and the consistency or their appearance both between fertile men and between individual ejaculates suggests that mature sperm RNA content is conserved and not a result of random retention. What then is the role of these retained RNAs? Studies have shown that the paternally derived RNAs are unique to the sperm cell and that, following fertilization, these same transcripts can be detected in the developing zygote (Hayashi et al., 2003; Ostermeier et al., 2004). To further establish the role of RNA delivered at fertilization in the epigenetic regulation seen early in zygotic development RNAs from various cell types were microinjected into the fertilized egg which resulted in a mutant phenotype (Rassoulzadegan et al., 2006). This data is further supported by the recent discovery of small nuclear and cytoplasmic RNAs found in fertile men (Krawetz et al., 2011). These data suggest that small miRNAs and piRNAs may play a role in ensuring the compatibility of the two genomes immediately following fertilization. From these data it appears that there is a role for both paternal ncRNA and mRNA transcripts in early embryonic development, although the specific roles for each has yet to be elucidated.

## CONCLUSION

Although the role of the paternal epigenome in embryogenesis has yet to be fully established, many important factors have been described and remaining questions are becoming more clearly focused. It is apparent that the selective retention of histone in the mature sperm likely has programmatic implications in early embryonic development, based on the localization and pattern of modifications of the marks. The process of protamine removal and replacement with maternal histones is not well understood, but is clinically very interesting since it may highlight the interaction of pre-fertilization and post-fertilization epigenetic remodeling events in the formation of the embryonic genome. Additionally, de-protamination and resetting of the male pronucleus epigenome may highlight another potential mechanism by which sub-optimal oocytes may affect embryogenesis. In other words, it may be possible that the oocyte has inherent corrective ability of sperm epigenome defects that are variable dependent on the overall oocyte “quality.” Lastly, it is becoming more clear that the RNA transcripts delivered to the oocyte via the sperm are important in the early embryo development, but at this point our understanding is in early infancy. What we have learned from preliminary studies is intriguing, but it requires further investigation.

The difficulty in obtaining samples for study has limited our ability to generate the required data to fully elucidate the roles of both the maternal and paternal epigenome in post-fertilization development and beyond. Increases in our knowledge regarding the biology of reproduction will require innovative ideas and techniques. The establishment of a more complete mechanistic picture of gamete fusion, and early embryonic development will provide fundamental knowledge that will improve diagnosis and treatment options for those individuals suffering from various forms of infertility.

## Conflict of Interest Statement

The authors declare that the research was conducted in the absence of any commercial or financial relationships that could be construed as a potential conflict of interest.
